# An integrated approach for the restoration of Australian temperate grasslands invaded by *Nassella trichotoma*

**DOI:** 10.1038/s41598-022-25517-3

**Published:** 2022-12-09

**Authors:** Talia Humphries, Christopher Turville, Steven Sinclair, Singarayer Florentine

**Affiliations:** 1 The Future Regions Research Centre, School of Science, Physiology and Sport, Federation University Australia, Mount Helen, VIC Australia; 2 Faculty of Science and Technology, Federation University Australia, Mount Helen, VIC Australia; 3 Department of Environment, Land, Water and Planning, Arthur Rylah Institute, Environment and Climate Change, Heidelberg, VIC Australia; 4 Applied Chemistry and Environmental Science School of Science, STEM College, RMIT University, 124 La Trobe St, Melbourne, VIC 3000 Australia

**Keywords:** Environmental impact, Environmental sciences

## Abstract

Invasive plants are considered to be one of the biggest threats to environmental assets, and once established, they can be immensely difficult to control. *Nassella trichotoma* is an aggressive, perennial grass species, and is considered to be one of the most economically damaging weeds to grazing systems due to its unpalatability, as well as being one of the leading causes of biodiversity loss in grassland communities. This species produces high density seedbanks that rapidly respond to disturbance events. Despite control programs being developing in Australia since the 1930s, this species is still widespread throughout south-east Australia, indicating that a new management approach is critical to control this Weed of National Significance at the landscape scale. The present study explored the effect of 12 different combinations of herbicide, fire, a second application of herbicide, grazing exclusion, tillage and broadcasting seeds in order to reduce the above and below-ground density of *N. trichotoma*. A control treatment was also included. The results were assessed using a Hierarchy analysis, whereby treatments of increasing complexity were compared for their efficacy in reducing *N. trichotoma* cover and seedbank density, while simultaneously increasing the establishment of the broadcast species. Whilst all integrated treatments effectively reduced *N. trichotoma*’s seedbank, the treatments that included fire performed significantly better at simultaneously reducing *N. trichotoma* and increasing the establishment of broadcasted seeds. Overall, the integration of herbicide, fire and broadcasting native seeds was observed to provide the most economically feasible management strategy for the landscape scale restoration of a degraded temperate grassland dominated by *N. trichotoma*.

## Introduction

Invasive plant species are considered to be as one of the most important threats to environmental assets^1–3^, which includes such essential holdings as grassy ecosystems. Once established, invasive species can (i) alter the quality of the pasture available for grazing, (ii) change the fire regime characteristics of the above-ground material^4,5^, (iii) degrade the soil quality^6,7^, and (iv) modify the soil’s hydrological processes^8,9^. Together, these pressures can cause significant environmental state changes, often leading to reduced habitat^10^ and altered food webs for higher tropic levels^11^, which result in severe changes in biodiversity and ecosystem functionality^12^.

The perennial grass *Nassella trichotoma* (Nees) Hack. ex Arechav. (serrated tussock) is considered to be one of the most destructive invasive grass species in Australia^13^, South Africa^14^ and New Zealand^15^. It is also an emerging weed in both the USA, where it is considered to be a Federal Noxious Weed^16^ and several West European countries^17^. It is considered to be amongst the most economically damaging weed species in Australia and New Zealand, with conservative cost estimates of $AU 40.3 million^18^ and $NZ 27.1 million^19^, based on reported costs of control procedures and loss of production. In South Africa, particularly within the Eastern Cape (Karoo), the control cost often exceeds that of the economic potential of the land, resulting in land abandonment^20^.

*Nassella trichotoma* is native to South America, but and has evolved multiple biological and ecological strategies that give it a substantial competitive establishment advantage which makes it difficult to control in many environments. It possesses a dense tussock growth form that acts to protect the plant’s base from frost and fire damage^21^, allowing the plant to survive severe climatic events. In addition, moderate fire events have also been observed to promote its germination^14,22^. Whilst this tussock grass normally requires between 500 to 900 mm of annual precipitation for optimum growth^23^, adult plants are tolerant to osmotic stress in times of moisture scarcity^21,22^. This is attributed to this species having (i) a shallow and fibrous root-system that allows for effective moisture uptake in areas of sporadic rainfall and (ii) tightly rolled leaves, which act to reduce transpiration^24^. It is therefore common to observe populations of this weed in areas that receive below optimal rainfall (< 500 mm annually)^25^. With regard to its physical properties, the leaves are high in fibre and extremely low in protein, making this grass unpalatable to grazing animals^21,26^, leading to it avoiding grazing pressures^27^. An adult plant’s canopy cover reaches approximately 50 cm in diameter, and together with its dense population, it means that the leaves of individual plants overlap, effectively shading out competing species^22^. Additionally, this perennial grass lives for up to 20 years^22^, and each plant can produce in excess of 100,000 wind-dispersed seeds per year^21^. Due to the prolific seeding ability of this species coupled with its anemochory seed dispersal, it can rapidly develop dense seedbanks, despite the majority of the seeds persisting for less than one year^28,29^. When taken together, these properties make *N. trichotoma* a very aggressive exotic grassland and pasture weed, which also effectively resists many attempts at its control.

Testament to the importance of this problem is that management efforts for dealing with *N. trichotoma* have been developing since the 1930s in Australia^30,31^, South Africa^32^ and New Zealand^33^. In each of these locations, *N. trichotoma* was unintentionally introduced and then become widespread. The most widely used control tactic currently used in Australia is spraying with the herbicide fluropropanate, which can be used to semi-selectively kill *N. trichotoma* since many Australian native grasses are not significantly harmed^34^. This residual herbicide provides effective control of *N. trichotoma* for up to five years before follow up treatments or reapplication is required^21,35^. While this herbicide has been successful for reducing *N. trichotoma* in some parts of Victoria, fluropropanate is not suitable for all landscapes and, in many cases, the weed returns to the site once the effects of the herbicide have worn off. This throws into question its suitability as a long-term solution^36^. Whilst this herbicide provided similar efficacy in South Africa, lower doses were recommended in order to maintain a high native *Eragrostis* spp. cover^37^. Native and introduced grasses were reduced in New Zealand by fluropropanate, and consequently it is not recommended for boom-spraying^38^. Rather, manual removal via grubbing has been found to be more efficient, and therefore this is widely used as a control method in New Zealand^39,40^.

These aggressive biological and ecological attributes of *N. trichotoma* directly contribute to the loss of ecosystem function to invaded ecosystems. There are significant challenges associated with controlling this weed at the landscape-scale, since once established, *N. trichotoma* forms a self-facilitating negative feedback loop that excludes favourable grasses. This is further complicated by the likeliness that areas where *N. trichotoma* has been abundant for some years will have passed biotic and abiotic ecological thresholds, and thus active intervention is required to restore ecosystem structure and natural processes^41^.

In addition, the natural migration of native seeds from surrounding areas is also limited by landscape fragmentation and scarcity of remnant grasslands, suggesting that relying on natural recruitment of native species in invaded grasslands is not a feasible strategy^42–44^. As a result of this adjacent seed deficit, implementing seed broadcasting, as an integral part of the weed management process, is likely to be important to the long-term reduction of invasive plants such as *N. trichotoma* which are poor competitors in their juvenile stage^21,22^. In this situation, it is suggested that management techniques should not only engage in the removal of above-ground *N. trichotoma* mass, but also improve abiotic conditions for the establishment of desirable and competitive grass species^45^, particularly during the early stages of development of *N. trichotoma*.

The complexity of the problem suggests that *N. trichotoma* control will require approaches that deal with multiple aspects of the invasion, including above ground weed biomass, native species biomass and soil seed banks, and this will be interlinked with ecosystem restoration strategies to return functional traits to the degraded grassland. The present study uses field trials to investigate the effect of integrating a series of available control tactics for this purpose. An increasingly integrated series of proven control actions has been sequentially tested for *N. trichotoma* control under the aegis of a Hierarchy analysis (Table 1). The control actions that were selected are globally available^46^, allowing the findings of this research to also have implications for the management of this weed in invaded locations outside of Australia.

**Table 1:** The 13 integrated treatment combinations and the associated treatment codes.

Treatment number	Treatment combination	Treatment codes
1	Control (no treatment)	NT
2	Herbicide + Seed Addition	HS
3	Herbicide + Grazing Exclusion + Seed Addition	HGS
4	Herbicide + Tillage + Seed Addition	HTS
5	Herbicide + Second Herbicide + Tillage + Seed Addition	HH*TS
6	Herbicide + Grazing Exclusion + Tillage + Seed Addition	HGTS
7	Herbicide + Grazing Exclusion + Second Herbicide + Tillage + Seed Addition	HGH*TS
8	Herbicide + Fire + Seed Addition	HFS
9	Herbicide + Fire + Grazing Exclusion + Seed Addition	HFGS
10	Herbicide + Fire + Tillage + Seed Addition	HFTS
11	Herbicide + Fire + Grazing Exclusion + Tillage + Seed Addition	HFGTS
12	Herbicide + Fire + Second Herbicide + Tillage + Seed Addition	HFH*TS
13	Herbicide + Fire + Grazing Exclusion + Second Herbicide + Tillage + Seed Addition	HFGH*TS

The objectives of this Australian-based research were to (i) find a cost-effective, long-term, integrated method for reducing *N. trichotoma*’s above-ground cover as well as its seedbank density, (ii) identify which of these control methods best enhances the establishment of two Australian native grasses, and (iii) as a result of these findings, make recommendations of how these methods could be adapted by land managers to treat similarly affected grasslands in other global areas. Consideration of the described ecological and biological traits of *N. trichotoma*, coupled with its long-term dominance at the study site, led to the hypothesis that the combination of herbicide and fire will effectively kill standing plants and reduce the seedbank through either devitalizing the shallow buried seeds, or through flushing the seedbank by triggering a mass germination response to the disturbance event. In the case where the seedbank is stimulated by fire, the subsequent treatment of a second herbicide application will reduce the establishment of the emerging seedlings. We intended that these combined treatments would allow for the broadcast native species to establish and flourish under low competition conditions. We also hypothesise that tillage will soften the soil to allow more effective establishment for the broadcast seeds, and the grazing exclusion treatment will reduce grazing damage from Macropods, subsequently, enhancing their successful establishment.

## Methods

All methods used in this field trial were performed in accordance with the university research guidelines and regulations.

### Site description

The study took place at Little Raven, located in Mambourin, Victoria, Australia (37° 55′ 18.12″ S, 144° 32′ 43.079″ E), west of Melbourne on the Werribee Plains. This site has an average annual rainfall of 468 mL, with the highest rainfall occurring during Autumn. The highest average temperature of 26 °C occurs in January, and the average low of 5 °C occurs in July^47^. This site has a history of sheep grazing, which facilitated the encroachment of invasive grasses through the reduction of native vegetation, such as *Themeda triandra*, the cover of this species being now depleted regionally due to grazing. In addition, a vegetation survey conducted by the Department of Environment, Land, Water and Planning^48^ identified that 54 different weed species belonging to 20 different families had established within this site, with the most notable dominant species being *N. trichotoma*.

### Site setup

Experimental plots were established in areas that had high densities of *N. trichotoma* (80% or greater foliage cover), which had not been recently treated with herbicide. A total of 78 plots measuring 10 × 10 m were established and marked with metal tags. Each plot was separated from the next by a 1 m buffer of untreated area. The 36 fire-treated plots were clustered in the south of the site, while the remaining 36 treatment plots and six no treatment (control) plots were clustered together in the north-east of the site. These plots were grouped together (i) to ensure that the fire and fire parameters including smoke and radiant heat, did not interfere with the control plots, and (ii) to make the large-scale burn manageable. Within the two locations, treatments were randomly assigned to each plot.

### Soil analysis

To provide data for comparison, eight soil cores were collected at random locations within the site. Samples were taken prior to implementing treatments at the Little Raven site. The soil analysis was conducted by the CSBP Soil and Plant Analysis Laboratory, and the Cowell Method was used to analyse phosphorous and potassium as described in Cowell^49^. The results of the soil tests were compared to those collected from a reference site, which is a nature conservation reserve located at nearby Mt. Cottrell. This reference site is approximately 20 km from Little Raven and shares the same soil type and original vegetation. The data was analysed using ANOVA on Microsoft Excel.

### Description of treatments

The treatment activities selected for this work are:

(i) herbicide spot-spraying with glyphosate to destroy all above-ground matter, treatment code H;
(ii) seed addition using native Wallaby-grasses (mixed *Rytidosperma* spp.) and Slender Spear-grass (*Austrostipa scabra* subsp. *falcata*) seeds of local provenance, treatment code S;
(iii) burning of residual above ground matter, treatment code F;
(iv) establishment of fencing to prevent grazing damage to the broadcast seeds, treatment code G;
(v) second respraying with glyphosate to remove sprouted *N. trichotoma* from the seedbank, treatment code H*;
(vi) tillage to bury *N. trichotoma* seeds and prepare the soil for the broadcast species, treatment code T.

In total, there were 12 integrated treatment combinations, plus a control treatment that received no management, which provided data for the Hierarchical analysis. Table 1 lists the treatments and provides treatment codes for the integrated treatments used throughout this work.

For treatment element H, all *N. trichotoma* plants were spot-sprayed using a backpack spot-spraying applicator with the recommended rate of glyphosate (100 mL to 10 L of water). Each plant was thoroughly sprayed to ensure all leaves and the bases of the plants were covered. Using this method, the plots selected to be fire treated were sprayed on the 25th of September, 2018, to allow sufficient time for the plants to dry out before implementing the fire treatment. Due to the weather conditions, the unburnt plots were sprayed over two days; half on the 31st of October and the other half on the 17th of December, 2018.

The fire treatment, F, was implemented on the 4th of December, 2018 by Parks Victoria and the Victorian Country Fire Authority (CFA).

The treatment by Grazing exclusion, G, was achieved by fencing each selected plot by securing ‘stocklock’ wire fencing to 150 cm stock fence posts. While the site is no longer used for livestock grazing, Eastern Grey Kangaroos (*Macropus giganteus*) are frequently observed grazing at the site. High visibility bunting was secured to the top of the fence post to deter kangaroos from jumping the fences of the plots. The fencing was installed in the unburnt plots during late November, and in January for the fire treated plots, after the burn had been conducted.

For the second herbicide application H*, selected plots were thoroughly examined and any emerging *N. trichotoma* plants were sprayed with the glyphosate solution, at the same concentration and application method as the first application, on the 8th and 9th of March, 2019.

Selected plots were tilled, code T, between the 17th and the 23rd of May, 2019, using a hand-held rotary hoe to depth of approximately 5 cm.

For the seeding treatment, S, *Rytidosperma* spp. and *Austrostipa scabra* seeds of local provenance to Little River were purchased from *Seeding Australia* and transferred to Federation University, Australia. These native species are both C_3_ grasses, and share a similar growth period to *N. trichotoma,* which offer a higher level of competition^50^. For each plot, a seed mixture consisting of 160 g of *Rytidosperma* spp., and 40 g of *A. scabra* was weighed and combined into paper bags. To prepare the seeds for broadcasting, the bagged seeds were mixed with sawdust to reduce the seed clumping due to the intertwining of awns. The seeds were broadcast between the 25th to the 31st of May, 2019, being scattered by hand, and then raked lightly into the soil with a garden rake.

### Data collection

#### Above-ground vegetation

The above-ground vegetation was surveyed during five sampling periods noted in Table 2, using point intercept transect lines^51^. For each plot, five evenly spaced 10 m transect lines were surveyed at 0.5 m intervals, starting at 0 m and ending at 10 m. A 0.5 m interval was selected as this length is slightly wider than a typical *N. trichotoma* plant. A wire pole was placed on the ground at each 0.5 m point, and the vegetation that it touched, inclusive of plant base, leaves or flower heads, was identified and recorded.

**Table 2:** The dates that each sampling period was undertaken for the above-ground vegetation surveys.

Sampling period	Start date	End date
1	28/08/2018	16/10/2018
2	4/02/2019	18/02/2019
3	12/12/2019	24/01/2020
4	2/11/2020	20/11/2020
5	28/9/2021	18/10/2021

#### The seedbank

To sample the seedbank, a soil corer (5 cm diameter and 5 cm depth) was used to collect cores from the centre, and then from three randomly selected locations within each plot. The four soil samples were combined into a zip lock plastic bag, labelled with the plot’s tag number and then transported to a glasshouse at Federation University Australia, Mt Helen. The bags were left open to allow the soil to air dry for two weeks. Plastic punnets (14 cm × 8 cm × 5 cm) were prepared by placing a sheet of absorbent towelling at their base, and then adding 50 g of river sand and 160 g of the collected dry soil. The sample from each plot was divided into five replicates, with two punnets from each plot randomly selected to be initially watered with 10% smoke water solution and then subsequently only with tap water. The smoke was used to stimulate germination of species with germination cues associated with fire parameters. The punnets were placed into butcher’s trays (28 cm × 44 cm × 5.5 cm) to allow bottom watering. Tap water was added to the butcher’s trays three to five days a week, and emerging seedlings were identified and removed weekly. Seedlings that could not be identified immediately were replanted separately and grown until identification could be made with confidence. Soil cores were taken for the pre-treatment survey on the 13th and the 20th of August, 2018, and cores were collected from the fire plots on the 20th of December 2018 to assess the effect of the fire treatment on the seedbank. Two further sampling periods were conducted; one in 2019 and one in 2020. A research permit to handle *N. trichotoma *ex situ, as well as other nationally significant weeds that may be present within the soil seedbank, was obtained prior to taking soil cores. Permission from the land managers and cultural heritage partners was also granted prior to conducting this experiment.

#### Estimating costs of treatment application at different scales

In order to examine the efficiency of the various treatments, we estimated the costs (AUD) of implementing each of the treatments over areas of different sizes. To calculate herbicide application costs using backpack spraying, an unpublished cost model held by the Victorian Department of Environment, Land Water and Planning (DELWP) was used. This model considers labour and chemical costs, and assumes that effort is proportional to weed cover and site area (C. Hauser, DELWP, pers. comm.). To calculate the costs of implementing fire, we used unpublished cost estimates compiled by DELWP and Parks Victoria, that include planning, equipment and labour costs at pre-burn, burn and mop-up phases, and list examples for fires of different sizes (S. Sinclair, DELWP, pers.comm.). Cost estimates for tilling were based on the cost estimates that Parks Victoria use for ploughing. The cost for broadcasting the native seeds was based on the purchase cost for the seeds used in the present study.

### Data analysis

#### The hierarchy analysis

**Figure 1:**
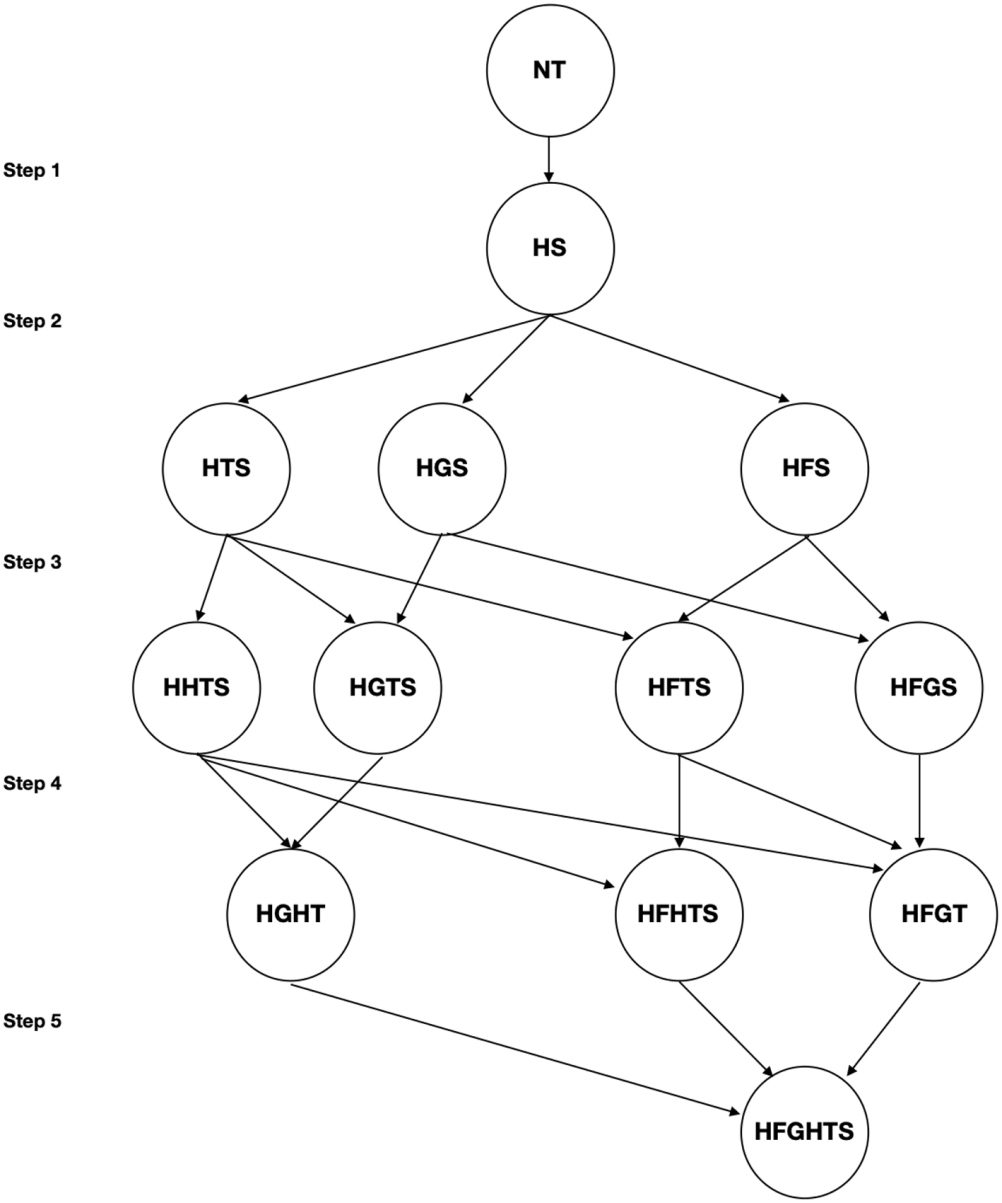
The hierarchy analysis process used to analyse the effect of the treatments on *N. trichotoma* and the establishment of the broadcast seeds. The treatment codes shown in the flowchart are defined in Table 1.

The collected data was organised using a spreadsheet to determine the cover of each species of interest; *N. trichotoma*, *A. scabra*, and *Rytidosperma* spp., for each treatment during each sampling period. Due to the volume of data involved, the analysis was strategically implemented using a hierarchy analysis, which allowed elimination of non-significant treatment combinations and their subsequent hierarchy paths (Fig. 1). This process assisted with understanding management implications, as it helped to highlight the most successful result with the least number of control methods. *Nassella trichotoma* cover, *N. trichotoma* seedbank density, *A. scabra* cover and *Rytidosperma* spp. cover, were analysed separately.

The flowchart followed the path of statistically significant (p = 0.05) reduction for *N. trichotoma* cover and seedbank density, and the conditions for significant increase of the broadcast species cover. At each step in the analysis, the changes in above-ground cover were analysed over five sampling periods (i) within each treatment on its own, and (ii) between each treatment within the given step of the analysis. The same process was performed for the seedbank density analysis over three sampling periods. When no significant results are observed between treatments, or the analysis reaches the final step, the analysis was complete. The analysis was achieved using the statistical program SPSS (IBM^®^) and running the selected data through a syntax file to test the study hypothesis using; (i) a pairwise comparison, (ii) a univariate test, and (ii) a mixed model analysis.

The flowchart presented in Fig. 1 shows the possible analysis pathways for the increasing complexity of integrated treatments used in this study. The analysis is conducted in a series of steps:

- *Step 1* compares the control (NT) to the simplest treatment combination used in this study; herbicide and seeding (HS). If the HS treatment was seen to produce a significant result, Step 2 was invoked.
- *Step 2* compares the HS treatment to the treatments that added fire (HFS), grazing (HGS) or tillage (HTS). If a significant result was observed in Step 2 of the analysis, the analysis continued to Step 3.
- *Step 3* follows the path of significance indicated by the flowchart. There are three possible paths for analysis, determined by what treatment produces a significantly better result:
  1. The HTS treatment is compared to the HH*TS, HGTS, and HFTS treatments,
  2. The HGS treatment is compared to the HGTS, and the HFGS treatments,
  3. The HFS treatment is compared to the HFGS and the HFTS treatments.
- *Step 4* again follows the path of significance with four possible pathways:
  1. The HH*GS treatment is compared to the HGH*T, HFH*TS, and the HFGT treatments,
  2. The HGTS treatment is compared to the HGH*T treatment,
  3. The HFTS treatment is compared to the HFH*TS and the HFGT treatments,
  4. The HFGS treatment is compared to the HFGT treatment.
- *Step 5* compares the significant treatment from Step 4, either the HGH*T, HFH*TS, or the HFGT treatment to the HFGH*TS treatment.

#### Analysis of the vegetation community

The total cover of each species was recorded within each treatment at each sampling period. The vegetation community data was analysed by categorizing the recorded species into one of three groups; (i) Australian native species, (ii) invasive grasses, or (iii) invasive “other”, which included invasive herbs, forbs and shrubs. This was done separately for each treatment at each sampling period. Cover was assigned and calculated for these groups, rather than for the individual constituent species. Figures were created using Microsoft Excel.

## Results

### Soil conditions

Table 3 presents the results of soil nutrient analysis, which suggests that soil nutrients at Little Raven are generally significantly higher than that at the reference site.

**Table 3:** Soil nutrients and other parameters of the Little Raven study site and the reference site. Significance was set to *p* = 0.05 and these values are in bold.

Soil parameter	Little Raven	Reference site	Significance
Texture	Clay-loam	Clay-loam	
Ammonium nitrogen	25.00	4.38	**0.05**
Nitrate nitrogen	90.25	8.63	0.09
Phosphorus (Colwell method)	28.38	10.63	**< 0.01**
Potassium (Colwell method)	807.25	419.38	**0.01**
Sulphur	14.90	5.58	**< 0.01**
Organic carbon	4.61	2.46	**< 0.01**
Conductivity	0.22	0.09	**0.02**
pH Level (CaCl_2_)	4.91	4.85	0.66
Ph Level (H_2_O)	5.84	5.88	0.84

The soil data for the reference site was provided by Steve Sinclair (Victorian State Government).

### The hierarchy analysis

The results of the hierarchy analysis for the cover of *N. trichotoma*, *A. scabra* subsp. *falcata* and *Rytidosperma* spp. plus the *N. trichotoma*’s seedbank density are shown in Table 4. More detailed figures regarding the steps of each analysis are available as supplementary data.

**Table 4:** The results of the hierarchy analysis using the developed flowchart (Fig. 1). Each species was analysed separately and the analysis of the effect of the treatment combination, sample period and the interaction of these factors are shown. Significance was set to *p* = 0.05.

Hierarchy step	Time	Treatment	Time × Treatment
***Nassella trichotoma***			
1	0.017	0.103	0.002
2	< 0.001	0.251	< 0.001
3	< 0.001	0.096	0.216
***Nassella trichotoma* seedbank**			
1	0.028	0.44	0.304
2	< 0.001	0.639	0.669
3	< 0.001	0.639	0.006
***Rytidosperma* spp.**			
1	0.105	0.451	0.140
2	< 0.001	< 0.001	< 0.001
3	< 0.001	0.477	0.980
***Austrostipa scabra***			
1	< 0.001	0.006	< 0.001
2	< 0.001	0.001	< 0.001
3	< 0.001	0.470	0.919

**Figure 2:**
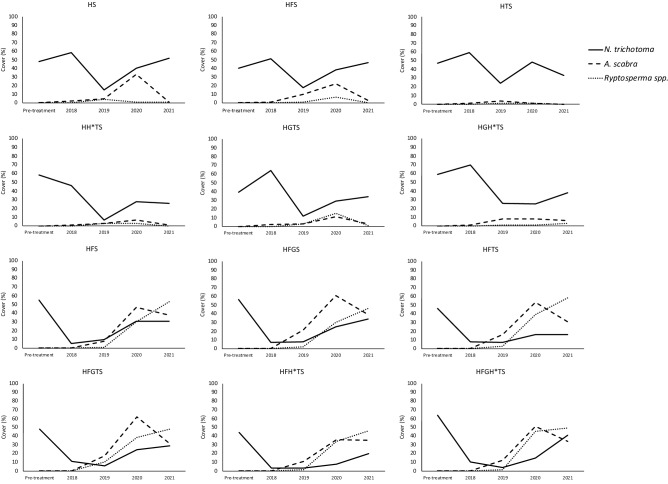
The mean cover (%) of *N. trichotoma* (solid line) and the two-broadcast species: *A. scabra* (dashed line) and *Rytidosperma* spp. (dotted line) for each treatment over the five sampling periods.

No step in the analysis observed a significant reduction in *N. trichotoma* cover at the final sampling period between any treatment (Fig. 2), despite significant reductions in cover between sample times within many of the treatments (Table 5). Time and the time-and-treatment interactions, however, were observed to be significant. When comparing the effect of the HS treatment with the control (NT), it was observed that the HS treatment caused a significantly lower (p = 0.002) above ground density of *N. trichotoma* compared to NT in 2019 (Fig. S1). The HS treatment was then compared to HGS, HTS and HFS treatments, where it was observed that the HFS had significantly reduced cover in 2018 (p < 0.001) compared to the unburnt plots. The HFS treatment was compared to the HFGS and the HFTS treatments, and it was found that the HFTS treatment was significantly better at reducing *N. trichotoma* cover than the HFGS treatment (p < 0.001), but not significantly better than the HFS treatment. No further significant differences were observed. The HFS treatment provided a significant reduction in *N. trichotoma* compared to the unburnt treatments during the 2018 sampling period, which was directly after the fire treatment. However, in the following sampling periods, *N. trichotoma* cover was not significantly different. Therefore, it was concluded that the HS treatment could provide the most effective solution for *N. trichotoma* control.

**Table 5:** The change in the mean *N. trichotoma* cover (%) from the first sampling period to the fifth sampling period for the treatments. The negative numbers signify a reduction of *N. trichotoma* cover, while positive numbers signify an increase in cover. Differences were considered significant when p < 0.05.

Treatment	Mean difference (%)	*p*-value
NT	+ 2.33	1
HS	+ 4.16	1
HTS	− 14	1
HH*TS	− 32.33	< 0.001
HFS	− 24.5	< 0.001
HFTS	− 30.33	< 0.001
HFHTS	− 19.67	0.26

The broadcast species were analysed separately, and, for both species, the effect of the fire treatment was a significant factor in their establishment (Fig. 2). For *A. scabra* (Fig. S2)*,* the time, treatment, and their interaction were significant (*p* < 0.001) in 2020 for the HS treatment compared to NT. The treatment and time factors, as well as their interaction were significant (p < 0.001) in the second step of the analysis, with HFS having the highest cover. The third step did not observe any significant interactions between the treatments. No significant difference was observed between the NT and HS treatments for *Rytidosperma* spp. (Fig. S3). Despite no significant results, the flowchart was followed to Step 2, where time, treatment and the interaction of these factors observed a significant result (*p* < 0.001). The HFS treatment had the highest cover of *Rytidosperma* spp. so Step 3 compared HFS, HFGS and HFTS, and no significant difference between the treatments was observed. For both of the broadcast species, HFS was determined to be the most effective treatment.

**Figure 3:**
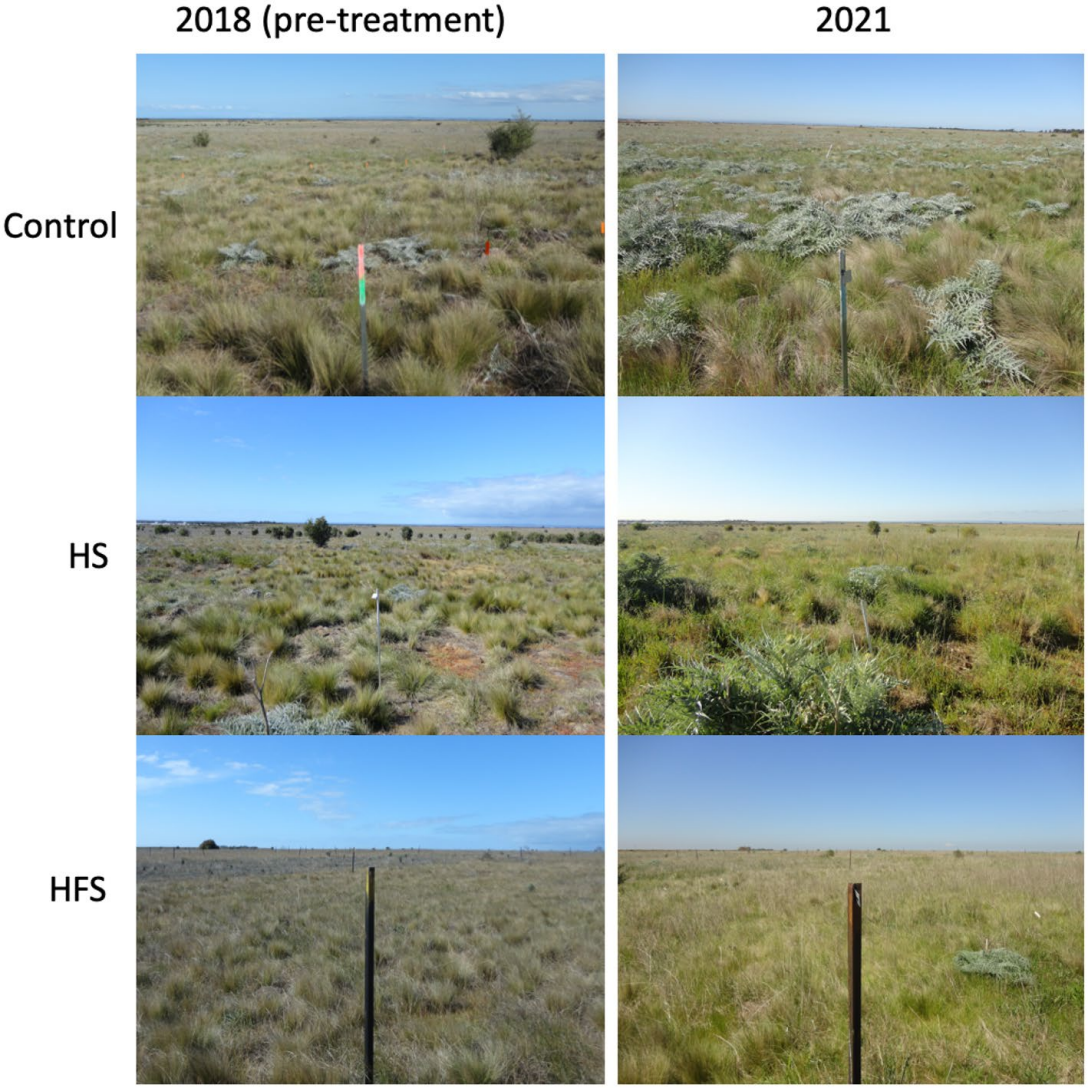
Photographs comparing the same plots at the first sampling period (left) and the fifth sampling period (right) for the HS and the HFS treatments*.* The control is included as a reference.

Figure 2 shows the changes in the three analysed species for each treatment over the five sampling periods. From this figure, it is evident that *N. trichotoma* cover is reduced most effectively by the fire and the till treatments. In 2019, one-year post-treatment, the cover of *N. trichotoma* was at its lowest across all the treatments. The broadcast species did not successfully establish in the unburnt plots, while all the fire treated plots experienced a significant increase in both species. Figure 3 shows photographs of the HS and the HFS treatments compared to the NT, (i) prior to implementing treatments (2018), (ii) and at the end of the final sampling period (2021).

**Figure 4:**
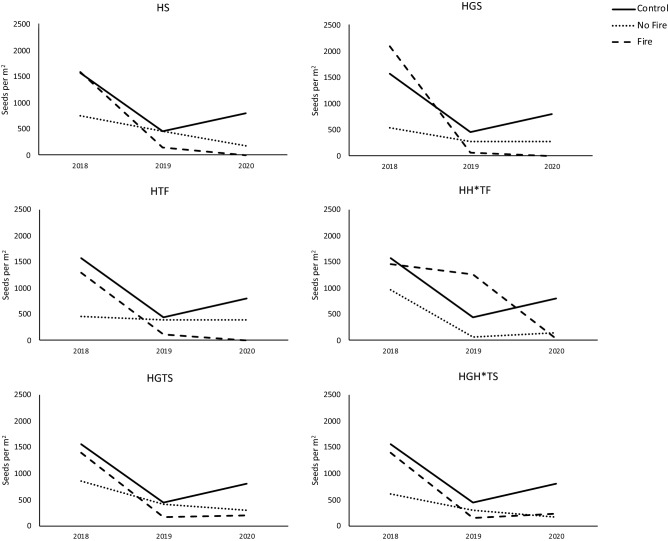
Changes in *N. trichotoma*’s seedbank over three sampling periods. Soil was collected pre-treatment in 2018, then post-treatment in 2019 and 2020. Each graph shows the control (no treatment) line for comparison (solid line), the unburnt plots are represented by the dotted line, and the fire treated plots are represented by the dashed line.

The analysis of *N. trichotoma*’s seedbank (Fig. S4) again, found no significant difference between the treatments for reducing the seedbank density, however significant reductions in *N. trichotoma*’s seedbank were observed between the three sampling periods across all the treatments (including NT) (Fig. 4).

The hierarchy analysis demonstrated that fire was the most significant factor for increasing the establishment of the broadcast species. For this reason, the vegetation community data focused on the effect of the treatments with and without fire for the native species, the invasive grasses, and the invasive other.

### Analysis of the vegetation community

**Figure 5:**
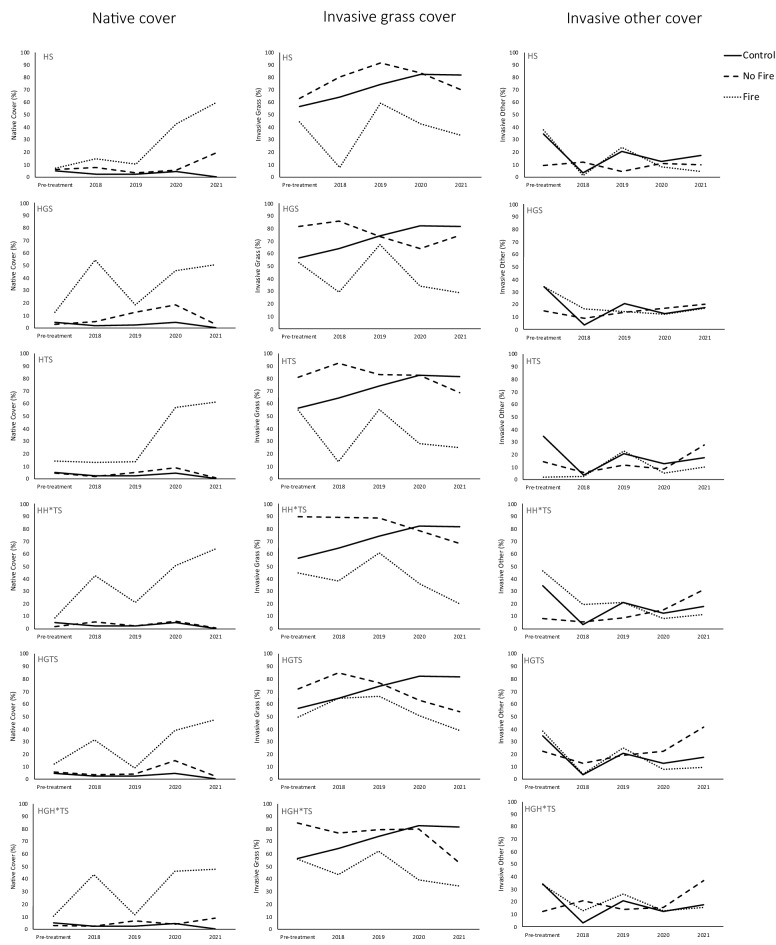
The effect of the treatments on the vegetation community mean cover of total native species (left column), total invasive grasses (centre column) and total invasive herbs, forbs and woody species (right column). Each row represents a different treatment, and the treatment code is listed beside each row (refer to Table 2). The control (NT) treatments are represented by the solid line, the unburnt treatments are represented by the dotted line, and the fire treatments are represented by the dashed line.

As fire was the most important factor at reducing *N. trichotoma* above and below-ground and also for increasing the cover of the broadcast species, we compared the effect of the treatment combinations on the vegetation community with or without fire. For this analysis, the vegetation community was divided into three categories; all observed native species were placed into one category, and the invasive species were categorised as either invasive grasses, or invasive other, which included invasive forbs, herbs and woody species. This analysis provides a general overview of the vegetation community as a result of the implemented treatments. The effect of the treatments, particularly fire, on the vegetation community, is visually shown in Fig. 5. In all cases, the fire treatment significantly increased the native species, particularly the broadcast species, compared to the same treatment without fire. The fire treatment was also observed to lower invasive grasses to a greater extent than that observed in the same treatment without fire. No treatments were effective at reducing the cover of the other invasive species, included forbs, herbs and shrubs.

In 2018, *N. trichotoma* was the most abundant species surveyed in all the plots (Table 6). In 2021, invasive grasses and invasive herbaceous species remained dominant for the unburnt plots, with the same invasive grass species; *N. trichotoma, Avena* spp., and *Nassella neesiana* being the top three species surveyed (Table 6). In the unburnt plots, the native broadcast grasses; *A. scabra* and *Rytidosperma* spp., did not successfully establish to provide effective competition, but rather, it was observed that there was an increase in invasive grasses including *Avena* spp. and *N. neesiana*. In contrast, the broadcast grass species were dominant in 2021 for the treatments that included fire (Table 6).

**Table 6:** The most abundant five species surveyed in the first and fifth sampling periods for the fire treated and fire excluded plots. The native species are highlighted in bold font.

Fire exclusion 2018	Fire exclusion 2021	Fire treated 2018	Fire treated 2021
*Nassella trichotoma*	*Nassella trichotoma*	*Nassella trichotoma*	***Rytidosperma***
*Avena *spp.	*Avena *spp.	*Romulea rosea*	***Austrostipa scabra***
*Nassella neesiana*	*Nassella neesiana*	***Austrostipa scabra***	*Nassella trichotoma*
*Romulea rosea*	*Cynara cardunculus*	*Nassella neesiana*	*Nassella neesiana*
*Cynara cardunculus*	*Lolium rigidum*	*Avena *spp.	*Sonchus oleraceus*

### Cost estimate

We found that the costs for implementing different treatments varied greatly, and were different for areas of different size (Table 7). The herbicide application is the most expensive treatment to implement in areas 10 ha or larger. For the treatments that used a second herbicide application, the cost for the herbicide application was doubled. Due to the costs associated with controlled burns, fire is a more economical action for large scale areas. Tillage is not often a feasible management action in grasslands that maintain their rocky composition, unless conducted manually as done in the present study, making this technique better suited to small plot scale areas. While ploughing machinery could be used to generate a similar effect, this would only be a feasible option in de-rocked areas. Broadcasting seeds becomes increasingly expensive with increased landscape size. As the seeds were only observed to establish when used in conjunction with the herbicide and fire treatments, it would be important to consider using these treatments in parallel to increase the rate of establishment.

**Table 7:** An estimate of the cost to implement each individual control method from the plot scale to increasing size. These figures were then combined to estimate the cost of implementing each treatment over increasing size scales.

**Cost ($AUD) of action for areas of varying size (Ha)**						
**Individual action**	0.01	1	5	10	50	100
Spot spray	35	3453	17,265	34,539	172,648	345,295
Apply fire	23,000	23,500	24,000	25,000	32,000	40,000
Till soil	40	90	450	900	4500	9000
Broadcast native seeds	44	440	2200	4400	22,000	44,000
**Multi-action strategy**						
HS	79	3893	19,465	38,939	194,648	389,295
HTS	119	3983	19,915	39,839	199,148	398,295
HH*TS	154	7436	37,180	74,378	371,795	743,590
HFS	23,079	27,393	43,465	63,939	226,648	429,295
HFTS	23,119	27,483	43,915	64,839	231,148	438,295
HFHTS	23,154	30,936	61,180	99,378	403,795	783,590

## Discussion

### Effect of the treatments on the above-ground vegetation community

This investigation shows clearly that the combination of herbicide spraying, fire and the broadcasting of native seeds significantly increased the establishment of the broadcast native species as well as reducing the above and below-ground density of *N. trichotoma*. As a consequence, we recommended this approach to be the most effective treatment combination. This research demonstrated that establishing robust competition provides sustainable, long-term control of invasive species^52,53^. To this end, the pre-seed broadcasting fire treatment was the most important control factor since, in this study, it provided a competition release and facilitated the establishment of the broadcast seeds, whilst the seeds failed to establish competition in the identical unburnt treatments.

A reason the broadcast seeds were only successful in the fire treated plots could be attributed to the rapid nutrient cycling and decreased competition, which in turn increases light and solar heating to the soil surface^54–56^. Altered levels of soil nutrients, are often found in degraded landscapes, which significantly favours the dominance of invasive plants. The soil analysis at Little Raven indicated elevated soil nutrients. Fire has been observed to reduce elevated soil nutrients by volatising N, P and S, as these nutrients have low temperature thresholds^57,58^. Fire also adds charcoal to the soil^59^, and this can act as an important stimulant for the seed germination of many native Australian grassland species^60^. In the unburnt treatments, invasive annual species outcompeted the broadcast species.

Removing the biomass of invasive plants prior to broadcasting seeds is critical for improving establishment rates of broadcast plant propagules^61–63^. The fire treatment cleared not only *N. trichotoma*, but also reduced the cover of other invasive plants, and the broadcast seeds in these treatments thus faced lower competition than in the unburnt treatments. In the unburnt treatments, the dead *N. trichotoma* plants continued to occupy a high proportion of these plots despite the herbicide effectively killing them. The dead plants were slow to decompose, and subsequently continued for some time to reduce space and light for the broadcast grasses. This provided a competition advantage for invasive annual plants, such as *Avena* spp., which generally establish well in nutrient-rich and shady places^64^, and were observed to increase in cover in the unburnt treatments over the five sampling periods. In the fire-treated plots, the broadcast seeds had high rates of establishment and provided effective competition against *N. trichotoma* and invasive annuals.

Despite the implementation of fire providing significant reductions in *N. trichotoma* cover, the largest reduction was observed for the herbicide + second herbicide + tillage + seed addition (HH*TS) treatment, with an average cover reduction of 32.33% observed at the fifth sampling period compared to the first. It is important to note that this treatment was the only unburnt treatment to see a significant reduction in *N. trichotoma* cover, and unlike the fire treatments, the reason for the reduction was not due to competition from the broadcast native seeds, as these did not establish in this treatment. Whilst invasive grasses declined in the HH*TS treatments, other invasive vegetation increased, such as *C. cardunculus*, which had an average increase from four to 14 plants per plot from the first to the last sampling period. In other words, the success of HH*TS in reducing *N. trichotoma* was offset by the disadvantage of allowing other invasive species to prosper.

Tillage was used to soften the soil, which was anticipated to assist the establishment for broadcast native seed, while also acting to reduce competition by burying the invasive seeds already present further into the soil profile. Tillage did not have a significant effect on the establishment of the broadcast seeds in the unburnt plots, nor did it significantly improve their establishment compared to the herbicide + fire + seed addition (HFS) treatment. Tillage provided better reductions in *N. trichotoma* cover, in both the burnt and the unburnt treatments, suggesting tillage buried the seeds in the topsoil to a depth that reduced seedling emergence. The second application of herbicide did not observe to improve *N. trichotoma* control, or establishment of the broadcast species.

The grazing exclusion was included to identify the effect of macropod grazing on the seed establishment. Fencing has also been used as a preventative strategy in *N. trichotoma* control to reducing seed immigration as the fence line can trap the panicles, thus reducing the spread of the seeds. However, the herbicide treatment applied in the present study prevented seed production of *N. trichotoma*, and the grazing exclusion treatment, therefore, did not significantly reduce the seedbank density compared to the unfenced treatments.

### Effect of the treatments on *N. trichotoma*’s seedbank density

All the treatments reduced *N. trichotoma*’s seedbank, including the control. While small numbers of *N. trichotoma* seeds are capable of long-term persistence, the majority of this species’ seeds germinate or decay within the first year^28,65^. The timing of the treatments was critical for the prevention of seed addition in 2018, as seed set begins at the start of Summer for this species. Therefore, by applying herbicide in Spring, the plants failed to produce fresh seeds for the year. The fire treatment was implemented in early Summer, which not only stopped seed set from mature plants, but could have flushed the seedbank through the promotion of germination or devitalizing any remaining seeds^66,67^. The seeds rely on wind for dispersal, and the reduced seed production from the treatments surrounding the untreated control plots (NT) may have inadvertently resulted in the density reduction observed within these plots, despite these plots retaining a high cover of *N. trichotoma*. Due to the treatments only being applied within the plots, healthy *N. trichotoma* plants surrounded the plots and continued to set seed each year, therefore the seed density results reported in this study may be higher than if the treatments were applied uniformly across a larger site.

### Factors effecting economical management of *N. trichotoma*

The two key factors that will assist land managers in deciding what are the most economical treatments for implementation are; (i) the area of the site and, (ii) the percentage of rocky composition of the grassland. The size of the site is important when selecting an appropriate control strategy, because the cost of different strategies vary with the scare of the undertaking, which means that a strategy that may be recommended for small sites may be economically unfeasible for larger areas. Indeed, some treatments increase steeply in cost as site area increases, such as for herbicide delivered by backpack or seed broadcasting, while other treatments only increase relatively slightly as site area increases. Fire, for example, can cover a relatively larger area with increasingly little extra input. Notwithstanding this complexity, if the site was unsuitable for fire, for example because f nearby infrastructure, herbicide application may have to be used to provide some level f control for *N. trichotoma*’s seedbank. In this situation, because our results showed that the HS treatment was not effective at reducing *N. trichotoma* above-ground cover alone, it is recommended that follow up with tillage and spot-spraying of remerging seedlings is required to see a significant reduction (p < 0.001).

In this regard, the option to incorporate tillage would be determined by not only the size, but also the rockiness of the site. If the site was small but rocky, it could be tilled manually using the same technique as demonstrated in this study. However, this would not be a suitable solution for even small to moderate sized sites due to the intensity of physical labour required. Tillage would be economically feasible for areas that have been previously cropped, and thus no longer maintain a rocky composition since ploughing equipment is easily damaged by rocks, and manoeuvring through rocky terrain slows down the process and subsequently increasing the labour costs.

It would not be recommended to add seeds without the prior application of herbicide and fire, as this treatment was critical for their establishment and thus needs to be included in the budget. If fire is implemented in late-Spring or early-Summer, it can be effective for preventing *N. trichotoma*’s seed set for the year, as well as removing excess biomass of standing plants to allow establishment of native species^21^. However, the inclusion of herbicide would be required to effectively kill the adult *N. trichotoma* plants, as they are able to reshoot and grow following fire disturbance.

Acquiring large quantities of native seeds of local provenance for grassland restoration can prove to be difficult^68^, and this can be a limiting factor on the scale of weed invaded area that can be targeted for treatment. The literature suggests that if seeds are able to recruit to a site naturally, applying a combination of herbicide with fire^69^ or biomass removal^70^ can reduce weed cover and promote the passive succession of native plants. Therefore, to overcome limited seed availability, as well as the high associated costs involved in large scale re-vegetation programs, applying the HFS treatment within a strategically selected area of a site could provide a seed source for the surrounding, untreated area^71^. This could promote natural succession of the native species out of the treated area into the surrounding untreated areas.

### Recommendations for future research

In this study, the objective of broadcasting seeds was to provide direct competition with *N. trichotoma* and prevent this species’ re-establishment. Whilst this study used two C_3_, perennial species that provided excellent competition with the target weed, it is suggested that a more diverse mix, that included C_4_ native grasses and native herbs or forbs, could have provided better year-round control of non-target invasive species^72,73^. Native species other than the broadcast species were rarely detected, and a more diverse seed-mix could be considered to enhance species richness^52^. Often, low diversity seed mixes have been observed to encourage the natural succession of a vegetation community, but this may be dependent on the native seedbank, or the ability for native propagules to naturally migrate^52^. As many degraded grasslands are fragmented and isolated from remnant sites, native propagules cannot readily recolonize naturally through migration^53^. As many grassland species have short-term seedbanks, with sites that have been in a degraded, weed-dominant state for an extended period of time may not have sufficient viable seeds available for seedbank recruitment^74,75^. Therefore, changes in the vegetation community will likely require human intervention^72^.

The methods described in this paper are also transferrable to grazing systems within Australia, and investigations into competitive palatable pasture grasses of different diversities should be conducted. Further, in order to transfer these methods to international grassland communities, particularly South Africa and New Zealand where *N. trichotoma* is wide-spread, the species selected for the seed-mix should be adapted to reflect the management goals of these areas. The findings of this research advise support previous observations that at least one grass species with similar growth parameters to *N. trichotoma* should be included for effective competition^76^.

## Conclusion

This study suggests a tractable pathway for the control of *N. trichotoma,* while simultaneously restoring native species to degraded grassland. The combination of herbicide, fire and broadcasting seeds has been shown to provide a significant reduction in *N. trichotoma* cover and a significant increase in cover by the broadcast species. It therefore appears that these integrated control methods are clearly adaptable to international grasslands and grazing systems where this species is dominant and difficult to control.

## Supplementary Information

Supplementary Figure S1.

Supplementary Figure S2.

Supplementary Figure S3.

Supplementary Figure S4.

## Data Availability

The data that support this study will be shared upon reasonable request to the corresponding author.
