# Supplementary material for: An integrated approach for the restoration of Australian temperate grasslands invaded by Nassella trichotoma
Source: Sci Rep. 2022 Dec 9;12:21364. doi: 10.1038/s41598-022-25517-3 (PMC9734104; doi:10.1038/s41598-022-25517-3)
Supplement: Supplementary file 1 — Supplementary Figure S1. [file 41598_2022_25517_MOESM1_ESM.docx]

**Figure S1:** The result of the hierarchy analysis on *N. trichotoma* cover. The individual graphs show each step of the analysis until no further significant differences were observed between the treatments.
